# Geographic Disparities in Survival After Surgery for Metastatic Bone Disease: A Retrospective Analysis from a German Sarcoma Centre

**DOI:** 10.3390/cancers17223664

**Published:** 2025-11-15

**Authors:** Wolfram Weschenfelder, Paula Maria Nickl, Friederike Weschenfelder, Christian Spiegel, Karin Gabriela Schrenk, Thomas Ernst, Mark Lenz

**Affiliations:** 1 Department of Trauma, Hand and Reconstructive Surgery and Orthopaedics, University Hospital Jena, 07747 Jena, Germany; paula.nickl@uni-jena.de (P.M.N.); christian.spiegel@med.uni-jena.de (C.S.); mark.lenz@med.uni-jena.de (M.L.); 2 Comprehensive Cancer Centre Central Germany, Campus Jena, 07747 Jena, Germany; karin.schrenk@med.uni-jena.de (K.G.S.); thomas.ernst@med.uni-jena.de (T.E.); 3 Department of Obstetrics, University Hospital Jena, 07747 Jena, Germany; friederike.weschenfelder@med.uni-jena.de; 4 Department of Haematology and Internal Oncology, Clinic of Internal Medicine II, University Hospital Jena, 07747 Jena, Germany

**Keywords:** cancer, metastases, metastatic bone disease, socioeconomic status, health service, prognosis

## Abstract

**Simple Summary:**

Metastatic bone disease is an important challenge in orthopaedic oncology, as improved cancer survival leads to more patients requiring surgery for skeletal stabilisation. Prognostic factors such as tumour type or pathological fractures are well known. Still, the influence of socio-economic determinants is less clear and has mainly been studied in the United States. In this retrospective analysis of 243 patients treated surgically for metastatic bone disease at a German sarcoma centre, we examined the relationship between socio-economic characteristics and postoperative survival. Socio-economic data were derived from patients’ place of residence and linked to national census indicators. While several variables showed associations in univariate analysis, only the size of the residential population remained significant in multivariate analysis. Patients from villages and large cities had worse survival compared with those from small or medium-sized towns. These findings suggest regional disparities and highlight the need to better understand geographic inequalities in cancer care.

**Abstract:**

Background/Objectives: Metastatic bone disease (MBD) poses an increasing challenge in orthopaedic oncology due to prolonged survival. While clinical prognostic factors are well established, the role of socio-economic determinants remains unclear, particularly within universal healthcare systems. Methods: We retrospectively analysed 243 patients who underwent surgery for MBD (excluding spine) between 2005 and 2024 at a German sarcoma centre. Socio-economic indicators were derived from national databases and linked to patients’ residential districts. Survival was analysed using Kaplan–Meier estimates and Cox regression, adjusting for clinical confounders. Results: Median postoperative survival was 22 months. Several socio-economic indicators—income, education, and employment—were associated with survival in univariate analysis. In multivariate models, only residential area size remained independently significant (*p* = 0.047). Patients from villages (<2000 inhabitants) and large cities (>100,000) had poorer survival than those from small or medium-sized towns. This effect persisted after adjustment for tumour type, pathological fractures, and year of surgery. Conclusions: Within a universal healthcare system, residential area size was associated with survival after surgery for MBD, suggesting that regional disparities may persist despite equal formal access to care. Further studies integrating individual-level socioeconomic data are needed to identify mechanisms and guide interventions to reduce geographic inequalities.

## 1. Introduction

With improvements in systemic cancer therapy leading to longer survival, metastatic bone disease (MBD) has emerged as a growing clinical challenge in orthopaedic oncology. It is estimated that up to 70% of individuals with advanced-stage breast, prostate, or lung cancer will eventually develop bone metastases [1,2,3,4,5]. These lesions often result in skeletal-related events (SREs), such as pathological fractures, bone pain, spinal cord compression, hypercalcaemia, and bone marrow suppression — all of which substantially diminish patients’ quality of life, functional capacity, and survival [1,6,7,8,9,10]. As a result, the clinical and socioeconomic burden of MBD continues to increase worldwide, with the economic impact in the United States alone having exceeded USD 40 billion in 2020 [6,11,12,13].

Among the malignancies most frequently associated with bone metastases are breast, prostate, lung, renal, and thyroid cancers. Prognosis in patients with bone involvement depends on various factors, including the number, distribution, and size of bone lesions, as well as the presence of pathological fractures. [3,5,8,14,15,16,17,18,19]. Patients with oligometastatic disease, typically defined as five or fewer bone metastases, often have a more favourable prognosis and may benefit from tailored multimodal therapeutic strategies [20,21,22]. In Germany, recent studies by Herget et al. and Raschka et al. have further characterised clinical and tumour-specific prognostic factors in surgically treated patients with bone metastases. Yet, these works did not include socioeconomic or geographic determinants of outcome [15,16].

Beyond tumour- and treatment-related factors, increasing evidence highlights the role of socioeconomic and structural determinants in shaping cancer outcomes. In the United States, several studies have demonstrated that insurance status, ethnicity, and residential context influence treatment access, adherence, and survival in patients with metastatic disease [23,24,25,26,27,28,29,30]. Similarly, Zamora et al. reported notable regional disparities in sarcoma care, particularly regarding access to specialised diagnostics and therapies [31]. However, evidence from European, and specifically German, healthcare systems remains scarce. Unlike the U.S. model, the German system provides universal healthcare coverage, but it remains unclear whether this structural equality fully mitigates geographic or socio-economic disparities in real-world oncologic outcomes.

The present study, therefore, aimed to explore whether regional and socioeconomic factors are associated with overall survival following surgery for metastatic bone disease within a universal healthcare system. Specifically, we assessed whether indicators of healthcare accessibility, educational and economic conditions, and population size at the place of residence were linked to survival differences among patients treated at a German sarcoma centre. By addressing these questions, this study provides one of the first analyses of social and spatial determinants of survival in metastatic bone disease within the context of a European healthcare system.

## 2. Materials and Methods

### 2.1. Study Population

**Figure 1:**
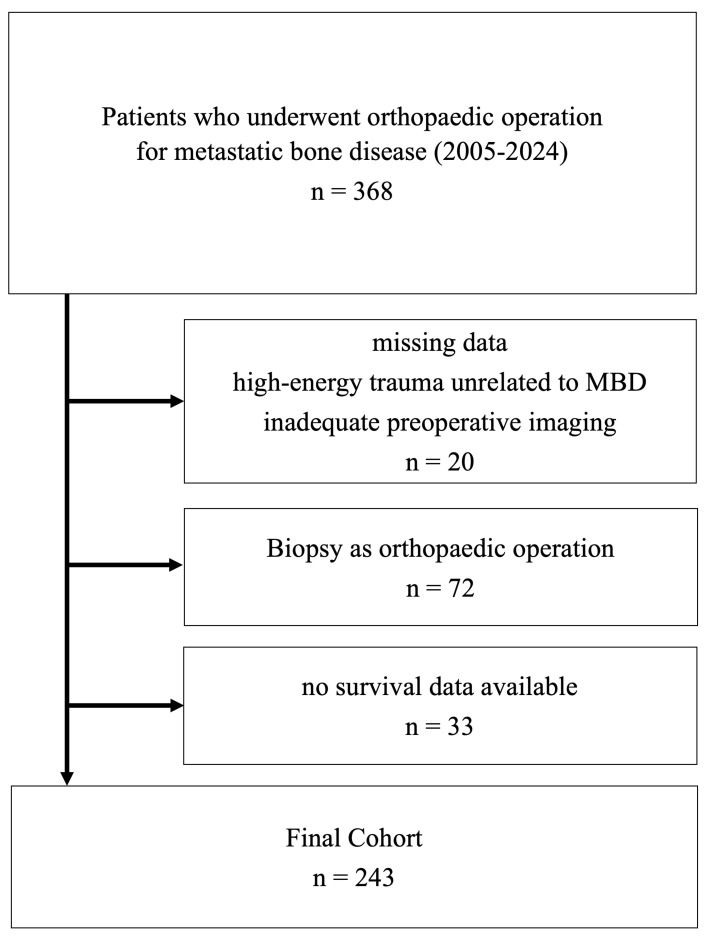
Flowchart of study population.

At the University Hospital Jena, we conducted a retrospective analysis of patients who underwent surgical treatment for metastatic bone disease, excluding spinal cases, between 2005 and 2024. Three hundred sixty-eight of the patients were eligible for inclusion. Twenty patients were excluded because of insufficient preoperative imaging, high-energy trauma unrelated to MBD, or incomplete data. Seventy-two patients were excluded because their operation was only a biopsy, and 33 patients had no complete survival data available. Two hundred forty-three patients were included in the final analysis (see Figure 1). Ethical approval was given by the local Ethical Committee of the Friedrich-Schiller-University, Jena, Germany (2023/3080-Daten).

### 2.2. Data Collection and Statistical Analysis

We extracted patient characteristics, clinical history, pathology findings, and imaging results from the medical records. Insurance status (statutory vs. private) was recorded; given the small number of privately insured patients (*n* = 22; 9%), comparisons were exploratory and assessed using Kaplan–Meier analysis with the log-rank test. Metastatic disease was categorised as single, oligometastatic (2–5 bone lesions), or polymetastatic (>5 bone lesions), whereas metastatic status was not evaluated in patients with lymphoma or multiple myeloma. The date of the confirmed last tumour follow-up or death of the patient was obtained from the Thuringian Cancer Registry, and survival status was calculated in months. Overall survival was defined as the time from surgery for metastatic bone disease to death or last follow-up.

Residential area size was determined using data from the state statistical office and categorised according to standard German spatial planning definitions: fewer than 2000 inhabitants (villages), 2000–20,000 (small towns), 20,000–100,000 (medium-sized towns), and more than 100,000 inhabitants (large cities). For multivariate analysis, these four categories were consolidated into two groups to achieve comparable group sizes: a “poor prognosis” group (villages < 2000 and large cities > 100,000) and a “good prognosis group (towns between 2000 and 100,000). This consolidation was data-driven to ensure statistical power, while remaining consistent with established urban–rural classifications.

District-level data on social determinants of health (SDOH) were retrieved from the INKAR database (Federal Institute for Building, Urban Affairs and Spatial Development, 2022). Following previous U.S.-based studies, selected indicators were grouped into domains reflecting accessibility, population structure, economic status, and healthcare provision. Accessibility measures included distance (in kilometres) to the treating sarcoma centre, travel time to the nearest regional centre, and degree of rurality. Population structure variables comprised mean age, population change, and the proportions of residents with higher education entrance qualification or without a school-leaving certificate. Economic indicators included unemployment rate, long-term unemployment, employment rate, median income, and the proportion of social welfare recipients. Healthcare access was assessed by the number of hospital beds per 1000 inhabitants and general practitioners per 10,000 inhabitants.

As SDOH indicators were available only at the district level, whereas population size was assigned at the municipality level, an ecological linkage approach was applied; potential inconsistencies between data levels are addressed in the Limitations section.

Statistical analyses were conducted using SPSS 28.0 (IBM©, New York, NY, USA). All eligible patients were included. Categorial variables were compared with the χ^2^ test, and continuous variables, which were non-normally distributed, using the Mann–Whitney U; medians with interquartile ranges are reported. Median survival was estimated using the Kaplan–Meier method and compared using the log-rank test or a Cox regression (omnibus test). Univariate Cox regression included 14 district-level variables. To control for type I error inflation, *p*-values were adjusted for false discovery rate (FDR) using the Benjamini–Hochberg method; both unadjusted and FDR-adjusted values are presented. Variables with *p* < 0.05 after adjustment entered the multivariate model. Independent predictors were identified using stepwise backward elimination (likelihood ratio method). Proportional-hazards assumptions were checked visually using log-minus-log plots for each key variable. No relevant deviations were observed.

To evaluate potential bias related to disease stage at presentation, landmark analyses were performed 6 and 12 months after diagnosis, including only patients alive at each landmark. Survival was re-estimated from each time point onwards.

## 3. Results

### 3.1. Baseline Characteristics

The study included 123 female and 120 male patients. Ages ranged from 35 to 91 years, with a median of 67 years (IQR: 59–73), and no significant differences were observed between genders (*p* = 0.41). Patients were initially diagnosed with cancer between 1995 and 2024. The median interval from primary cancer diagnosis to surgery for metastatic disease was 21 months (IQR: 1–77 months).

In 54 of 243 patients (22.2%), bone metastasis represented the initial presentation of cancer. Among these, 32 cases were classified as singular, 29 as oligometastatic, and 129 as polymetastatic. Of the 190 patients evaluated for visceral involvement, 99 (52.1%) had metastases beyond the skeleton. The most frequent primary tumours were renal cell carcinoma (53 patients), breast cancer (50), multiple myeloma (45), lung cancer (30), and prostate cancer (8), with the remaining 57 cases comprising a variety of other malignancies. Pathological fractures were identified in 153 patients (63%).

### 3.2. District-Level Characteristics and Social Determinants of Health

Among the 243 patients included in the study, 22 (9%) had private health insurance. No significant difference in survival was observed between the statutory and private insurance groups. Regarding marital status, 91 (37%) patients were single or widowed, while 152 (63%) were married. In terms of place of residence, 47 (19%) patients lived in villages with fewer than 2000 inhabitants, 87 (36%) in small towns with populations between 2000 and 20,000, 55 (23%) in towns with population size between 20,000 and 100,000, and 54 (22%) in cities with over 100,000 residents (see Table 1).

Detailed district-level variables and corresponding *p*-values from univariate Cox regression analyses of postoperative survival are presented in Table 2.

The overall median postoperative survival was 22 months (95% CI: 12–32 months), while the median survival from the time of primary diagnosis was 78 months (95% CI: 59–97 months). There was no statistically significant difference in survival between genders, with median postoperative survival of 26 months for women (95% CI: 9–43 months) and 16 months for men (95% CI: 8–24 months; *p* = 0.46).

Among the district-level characteristics, the size of the patient’s place of residence demonstrated a significant association with median survival (*p* < 0.001). Patients residing in towns with populations between 20,000 and 100,000 had the most favourable prognosis, with median postoperative survival not reached (12-month OS 63%; 95% CI 50–76%), whereas those living in cities with more than 100,000 inhabitants had the poorest outcomes, with a median survival of 12 months (95% CI: 5–19 months; see Table 1). As pathological fractures are known prognostic factors, their distribution across residence size categories was assessed; no significant differences were observed (*p* = 0.150).

Several metric district-level metrics were significantly associated with survival in univariate analysis, including driving time to the nearest regional centre, average age of the district population, population change over 10 years, educational attainment, employment rate, median income, number of hospital beds, and the number of general practitioners per 10,000 inhabitants (see Table 2). After adjustment for multiple testing using the Benjamini–Hochberg procedure, the same variables remained statistically significant.

**Figure 2:**
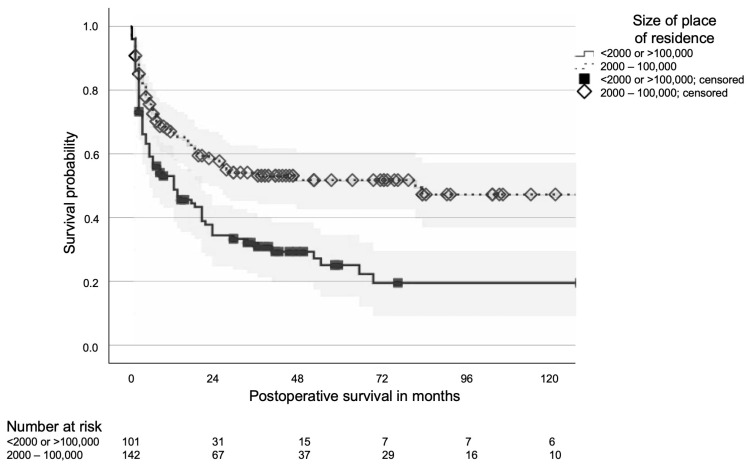
Kaplan–Meier curve of postoperative survival of patients operated for metastatic bone disease depending on the size of the place of residence; 95% confidence bands in grey.

For multivariate analysis, the four residence size categories were consolidated into a “good prognosis” group (residences with 2000–100,000 inhabitants) and a “poor prognosis” group (villages <2000 and cities >100,000 inhabitants, see Figure 2). In the initial multivariate Cox regression model including all variables significant in univariate analysis, residential area size remained borderline significant (*p* = 0.047; HR 1.61, 95% CI: 1.01–2.56; see Table 3). In a subsequent stepwise backward elimination model, residential area size was retained as the sole independent predictor and showed a strong association with survival (*p* < 0.001; HR 0.53, 95% CI: 0.38–0.74). When the year of surgery was added to the final backward elimination model, both variables (year and residential area size) remained independently significant (*p* = 0.003 each), confirming the robustness of the association.

To exclude bias from proximity-based fracture care or tumour-type–related survival effects, these factors were added to a separate multivariate model, where residential area size remained significantly associated with survival (Table 4). In the 6- and 12-month landmark analyses, including 218 and 192 patients, median overall survival increased from 22 months in the unadjusted cohort to 33 and 54 months, respectively. Prognostic factors remained unchanged, with residential area size persisting as the only independent variable in the multivariate Cox model.

## 4. Discussion

This study suggests that socio-economic factors may be associated with postoperative survival of patients with metastatic bone disease within the German healthcare system. In our cohort of 243 patients undergoing orthopaedic surgery for bone metastases, the only socio-economic factor that remained statistically significant in the multivariate analysis was the size of the residential area. Patients from villages with fewer than 2000 inhabitants and large cities with over 100,000 inhabitants showed poorer postoperative survival compared with those from medium-sized towns. These findings should be interpreted as exploratory associations rather than causal relationships.

To our knowledge, this is the first analysis examining socio-economic and regional determinants of survival in patients with MBD in Germany. Previous evidence originates largely from the United States, where socio-economic disparities are frequently linked to differences in insurance coverage, race, or access to specialised care [25,26,27,28,29,30]. In our study, no significant survival difference was observed between privately and statutorily insured patients in the Kaplan–Meier analysis. However, the number of privately insured patients was small (*n* = 22), which limits statistical power. Importantly, unlike the U.S. healthcare system, treatment in Germany is generally delivered in the same hospitals by the same providers regardless of insurance status, which may reduce the influence of insurance type on treatment outcomes. Still, our results indicate that structural or contextual inequalities may persist even under conditions of formal healthcare equality.

Recent German studies, such as those by Herget et al. and Raschka et al., have identified tumour-related and clinical prognostic factors in bone metastases, yet did not assess socio-economic influences [15,16]. Our findings, therefore, extend this body of evidence by highlighting that regional factors—particularly residential area size—may also contribute to survival variation in MBD.

The observed U-shaped pattern, with poorer survival in both very small and very large communities, may reflect different underlying mechanisms. In large cities, delayed presentation or complex social dynamics could contribute to later cancer diagnosis or fragmented care. In contrast, patients from small villages may face limited access to healthcare infrastructure or longer travel distances. Medium-sized towns might offer a more balanced combination of accessibility and social cohesion, resulting in more favourable outcomes. These interpretations, however, remain hypothesis-generating.

The association between residential area size and survival persisted after adjustment for key clinical factors such as pathological fractures, tumour type, and year of surgery, suggesting that this effect is not explained by these variables. To further account for potential bias related to early versus late metastatic disease, landmark analyses at 6 and 12 months after diagnosis confirmed the same prognostic pattern, with residential area size remaining the only independent predictor. This consistency supports the robustness of our results.

### Strengths and Limitations

The single-centre design ensured uniform surgical procedures and consistent documentation, enhancing internal validity. The linkage of clinical data with district-level socio-economic indicators allowed a broader contextual analysis.

However, several limitations must be acknowledged. The moderate sample size and tumour heterogeneity may have limited statistical power to detect smaller effects. Socio-economic indicators were only available at the district level, while residential population size was assigned at the municipality level. This ecological linkage may lead to information dilution and should be interpreted as reflecting area-level rather than individual-level effects. Residual confounding by unmeasured social or clinical variables cannot be excluded. Continuous modelling of urbanicity was not feasible because only categorical population data were available, and these change over time. The consolidation of the smallest (<2000) and largest (>100,000) municipalities was therefore performed to ensure statistical stability and interpretability while maintaining consistency with established spatial classifications. Patients excluded due to high-energy trauma (e.g., motor vehicle accidents) experienced fractures unrelated to metastatic bone disease and did not have increased early mortality; therefore, their exclusion is unlikely to introduce survival bias. Data on patients with MBD fractures managed non-operatively were not available, preventing sensitivity analyses including all MBD fracture admissions (intent-to-treat). Future multicentre studies using patient-level socio-economic data and larger cohorts are warranted to validate and refine these exploratory findings.

## 5. Conclusions

In this cohort of surgically treated patients with metastatic bone disease, residential area size was independently associated with postoperative survival, whereas other socioeconomic indicators were not. Patients from both small villages and large cities showed poorer outcomes compared to those from small and medium-sized towns.

These results suggest that regional disparities in outcomes may exist even within a universal healthcare system. Further research should clarify the mechanisms underlying these associations and identify strategies to improve equity in cancer care.

## Figures and Tables

**Table 1:** Ordinal healthcare research data of patients.

	Number (*n* = 243)	Postoperative Survival in Months (95% CI)	*p*-Value
Private insurance Public insurance	22 221	47 (0–96) 20 (12–28)	0.55
Size of place of residence (inhabitants)			<0.001
<2000	47	13 (0–30)	
2000–20,000	87	36 (0–84)	
20,000–100,000	55	not reached (12-mo OS 63%; 95% CI 50–76%)	
>100,000	54	12 (5–19)	
Size of place of residence (inhabitants)			<0.001
<2000 and >100,000	101	12 (3–21)	
2000–100,000	142	81 (n/a)	
Single/Widowed	91	26 (0–59)	0.47
Married	152	20 (4–36)	

*p*-value of Kaplan–Meier analysis with Log-rank-test; n/a not applicable; CI Confidence interval; for groups where median survival was not reached, fixed-time survival at 12 months is provided.

**Table 2:** Metric regional characteristics of places of residence.

	Median	Inter Quartile Range	Hazard Ratio (95% CI)	*p*-Value	FDR-Adjusted *p*-Value
Accessibility					
Distance to the treating sarcoma centre in km	43	18–85	0.997 (0.994–1.001)	0.20	0.26
Time to drive to the nearest regional centre in min	24.1	15.3–36.1	0.987 (0.977–0.997)	0.013	0.02
Rurality in %	44	0–60.3	0.994 (0.988–1.000)	0.071	0.098
Population					
Average age of district population in years	47.6	46.2–48.5	0.89 (0.83–0.96)	<0.001	0.012
Population changes within the last 10 years in %	−1.9	−5.5–1.4	1.07 (1.03–1.11)	0.002	0.014
Higher education entrance qualification in %	29.7	27.6–33.1	1.020 (1.007–1.034)	0.004	0.018
People without a school-leaving certificate in %	8.3	5.3–10.4	0.92 (0.86–0.98)	0.010	0.019
Economic situation					
Unemployment rate in %	5.4	4.5–5.8	0.98 (0.83–1.16)	0.80	0.80
Long-term unemployment rate in %	37.1	35.1–39	1.02 (0.97–1.08)	0.40	0.46
Employment rate in %	69.1	65.3–69.9	0.94 (0.90–0.98)	0.002	0.014
Median income in euros per month	2720	2610–2957	1.001 (1.000–1.001)	0.007	0.019
Recipients of basic income support in %	6.12	5.04–6.36	0.97 (0.89–1.05)	0.43	0.46
Local medical system					
Hospital beds per 1000 inhabitants	8.00	5.47–12.09	1.06 (1.02–1.11)	0.009	0.019
General practitioners per 10,000 inhabitants	13.81	12.76–19.01	1.06 (1.01–1.11)	0.011	0.019

*p*-values of Cox regression with omnibus-test of postoperative survival, FDR—False Discovery Rate.

**Table 3:** Multivariate survival analysis of significant healthcare parameters in univariate analysis.

Parameter	*p* Value	Hazard Ratio (95% CI)
Employment rate	0.27	0.93 (0.81–1.06)
Average age of the population of the district	0.37	0.85 (0.58–1.23)
Population changes over the last 10 years	0.45	0.94 (0.80–1.10)
Higher education entrance certification	0.25	0.96 (0.90–1.03)
People without a school-leaving certificate	0.162	0.91 (0.80–1.04)
Median income	0.55	1.000 (0.998–1.001)
Hospital beds per 1000 inhabitants	0.36	1.04 (0.96–1.12)
General practitioners per 10,000 inhabitants	0.42	1.08 (0.90–1.29)
Time to drive to the next regional centre	0.33	1.01 (0.98–1.04)
Size of the place of residence 2000–100,000 vs. <2000 combined with >100,000	0.047	1.605 (1.007–2.558)

Bivariate groups for size of place of residence used; CI: Confidence Interval.

**Table 4:** Multivariate survival model including size of place of residence, pathological fracture, and tumour type (CI: Confidence Interval, breast cancer as reference category for categorial variable tumour type).

Parameter	*p* Value	Hazard Ratio (95% CI)
Size of the place of residence 2000–100,000 vs. <2000 combined with >100,000	<0.001	1.86 (1.32–2.64)
Pathological fracture	0.009	1.65 (1.13–2.40)
Breast cancer	0.012	
Multiple myeloma	0.028	1.97 (1.08–3.60)
Renal cell carcinoma	0.029	1.95 (1.07–3.55)
Lung cancer	0.007	2.44 (1.28–4.67)
Other malignancy	<0.001	2.66 (1.53–4.63)

## Data Availability

The data presented in this study are not publicly available but are available on request from the corresponding author. The data are not publicly available due to privacy and ethical restrictions.

## Abbreviations

The following abbreviations are used in this manuscript:

AYAs	Adolescents and Young Adults
CI	Confidence Interval
CRP	C-reactive Protein
FDR	False Discovery Rate
IQR	Inter Quartile Range
MBD	Metastatic Bone Disease
SDOH	Social Determinants of Health
SRE	Skeletal Related Events
